# A systematic review of the associations between sedentary behavior, physical inactivity, and non-motor symptoms of Parkinson’s disease

**DOI:** 10.1371/journal.pone.0293382

**Published:** 2024-03-29

**Authors:** Aiza Khan, Joy Ezeugwa, Victor E. Ezeugwu

**Affiliations:** Faculty of Rehabilitation Medicine, University of Alberta, Edmonton, Alberta, Canada; University of North Texas Health Science Center, UNITED STATES

## Abstract

**Background:**

Parkinson’s disease (PD), known for motor symptoms, often presents early non-motor issues that significantly affect patients’ quality of life. While effective treatments are limited, physical activity and exercise offer potential benefits. However, an overlooked aspect of the movement intensity continuum is prolonged sitting or sedentary behavior, and physical inactivity. Thus, this study aimed to conduct a systematic review investigating the associations between sedentary behavior, physical inactivity, and non-motor symptoms, specifically cognitive impairment, depression, and poor sleep in PD.

**Methods:**

Conforming to PRISMA guidelines, a systematic search of the literature was conducted via electronic databases including MEDLINE, CINAHL, Scopus, PubMed and PsycINFO up to February 28, 2023. Studies were included if they investigated associations between sedentary behavior or physical inactivity and at least one non-motor symptom such as depression, poor sleep, and/or cognitive impairment, in adults aged 18 years or older with PD. Quality assessment of the studies was performed using the Newcastle-Ottawa scale for cross-sectional and cohort studies.

**Results:**

Of the 463 publications found, 7 studies met the inclusion criteria (n = 980 unique participants). Sample sizes ranged from 17 to 487 participants, and all studies were observational, conducted in home or community settings. Collectively, these studies show that higher amounts of both objectively-measured and self-reported sedentary time are associated with worse scores on standardized measures of cognition and the Parkinson’s Disease Questionnaire (PDQ) summary index and its subscales, such as cognition (memory and concentration). Additionally, longitudinal cohort studies suggest that physical inactivity and higher sedentary behavior are associated with depression and cognitive impairment in PD. Less sleep was associated with higher sedentary behavior.

**Conclusion:**

Associations observed between physical inactivity, sedentary behavior, and non-motor symptoms in PD underscore the need to address these factors for enhanced well-being. Further well-designed studies are essential to assess the impact of reducing sedentary behavior and physical inactivity on non-motor symptoms in PD.

**Prospero registration number**: PROSPERO (ID: CRD42023405422) on April 11, 2023.

## 1. Introduction

Parkinson’s disease (PD) is a progressive neurodegenerative disorder affecting 1 to 2% of adults over age 65 years and 4% of adults over age 80 years [1]. PD has been identified as the “fastest growing neurological disorder” between 1990 and 2016, contributing to a significant number of deaths and disabilities globally [2]. In 2019, PD was attributed with 5.8 million disability-adjusted life-years (DALYs) across 195 countries, representing an 81% increase since 2000 [2]. Clinically, PD is primarily characterized by motor symptoms such as bradykinesia, resting tremor, rigidity, gait abnormalities, and postural impairment [1]. However, it is important to note that a diverse range of non-motor symptoms (NMS) also play a significant role in PD symptomology [3, 4]. These NMS include sleep disturbances, sensory deficits, mood disorders including depression, apathy, or anxiety, autonomic nervous system dysfunction (orthostatic hypotension and obstipation), olfactory dysfunction, and cognitive impairment [3, 4]. Several studies indicate that NMS often precede motor symptoms and can have a more profound impact on the quality of life of individuals living with PD [5]. Mood disorders such as depression and anxiety, as well as pain, fatigue, and cognitive impairment contribute to the disease burden as PD progresses [3, 6].

Despite their significance, NMS frequently go unrecognized and undertreated, posing a challenge for people living with PD [7]. Moreover, NMS in PD may present significant challenges that affect day-to-day functioning and physical activity levels, as many people with PD often struggle to meet the recommended guidelines of 150 minutes per week of moderate-to-vigorous intensity physical activity [8]. In the general population, moderate-to-vigorous physical activity refers to activities performed at an absolute scale of >3 metabolic equivalents (METs), where a MET represents the energy equivalent that is expended while seated and resting [9]. For example, it is suggested that an individual can meet the moderate-to-vigorous physical activity threshold by walking briskly at 3–4 miles per hour and/or walking briskly uphill or while carrying a load [10]. In addition, Haskell and colleagues posited that an individual can meet the physical activity recommendation by walking briskly for 30 minutes two times a week and also engaging in a jogging program for 20 minutes on two other days [11].

With movement challenges and NMS experienced by people living with PD, moving fast or long enough to reach recommended targets may be hard. As a consequence, people with PD are physically inactive (i.e. an insufficient physical activity to meet the current physical activity recommendations [9] and many often lead sedentary lifestyle [12], which can have adverse short- and long-term effects on their health [13].

Sedentary behavior defined as “any waking behavior characterized by an energy expenditure of ≤1.5 METs, while in a sitting, reclining or lying posture [14], is more prevalent in individuals with PD compared to age-matched adults [15]. For example, people with PD engage in approximately 10 hours of sedentary behavior during waking hours, often in longer bouts compared to their age-matched peers without PD [15]. Notably, sedentary behavior is a well-established risk factor for chronic diseases such as diabetes, cancer, cardiovascular disease, and is negatively associated with mental health conditions like stress, anxiety, depression, and dementia [16]. Exploring the associations between sedentary behavior, physical inactivity, and NMS in PD may increase our understanding of the relationships between these behaviors to guide intervention development [3, 15]. Previous studies have shown positive effects of exercise on both motor and NMS in PD [17], however exercise (i.e. “a subset of physical activity that is planned, structured, and repetitive”) [18] and sedentary behavior are distinct and independent factors [15, 19]. Although a potential link between sedentary behavior and cognitive impairment has been suggested in PD [15], clear associations between other NMS, sedentary behavior, and physical inactivity are yet to be established.

In this paper, the authors aim to systematically review the evidence for the associations between sedentary behavior, physical inactivity, and NMS in individuals living with PD. Specifically, the authors focus on exploring the associations between sedentary behavior and physical inactivity with NMS related to mental health, including poor sleep, depression, and cognitive impairments.

## 2. Methods

### 2.1 Protocol and registration

In order to report this systematic review, we used Preferred Reporting Items for Systematic Reviews and Meta-Analyses (PRISMA) statement [20] as shown in S1 Table. The protocol was registered with PROSPERO (ID: CRD42023405422).

### 2.2 Identification of relevant studies

A systematic search was conducted to identify all original peer-reviewed articles available on the associations of sedentary behavior and physical inactivity with NMS, including sleep, depression, and cognitive impairment in PD until February 28, 2023. The search was performed on several databases, including MEDLINE, CINAHL, Scopus, and PubMed and PsycINFO using different combinations of search terms such as non-motor symptom* OR depress* OR anxiety OR mood AND/OR sleep* OR insomnia* OR sleep difficulties OR reduced sleep AND/OR cognitive decline, OR cognitive impairment OR sedentary AND sitting OR in-bed OR lying down OR physical inactivity OR lack OR minimal physical-activity, in different combinations with PD.

Next, all identified studies were transferred to Covidence software for screening.

The eligibility criteria were based on the PI(E)COS (participants, intervention (exposure), comparison groups, outcomes, and study design) framework [21]. The original peer-reviewed articles were included if they met specific criteria. First, studies were selected if they included participants with PD and at least one NMS was measured. Second, the exposure of interest was sedentary behavior or physical inactivity, as the primary focus of our study was to identify the association between sedentary behavior and physical inactivity with NMS. Regarding comparison groups, the comparison criterion was not applicable as all studies were observational studies. Patients from all settings, such as the community or home, were considered for inclusion in the study. In terms of outcomes, all the included studies measured at least one of the NMS, including depression, sleep, and cognitive impairment. Finally, all types of study designs were considered, as this is a relatively new area of study with limited available data. Therefore, all peer-reviewed articles written in English describing original quantitative research were included. Additionally, the reference lists of all eligible studies were carefully examined to identify any additional relevant studies.

### 2.3. Selection of relevant studies

Based on the eligibility criteria mentioned above, two independent reviewers screened the articles for selection. At first, the title and abstract screening was performed which was followed by the full-text screening. All conflicts between the two reviewers were discussed and resolved by the third reviewer and a consensus was reached.

### 2.4. Data synthesis

The data from the selected studies were extracted and organized using an Excel spreadsheet and Covidence software. The following information was collected: study number, title of study, first author, objective(s) of study, study design, year, country, number of participants, exposure setting, age, sex/gender, type of NMS measured and type of test used, measurement of sedentary behavior and physical inactivity and type of measurement used, main outcomes, secondary outcomes, key findings, and statistical analysis (available in S2 Table).

Due to factors such as small number of studies, variation in study designs and methods, and heterogeneity, it was determined that a meta-analysis may not yield worthwhile results. Therefore, a narrative synthesis was conducted, following the guidance by Popay et al. [22]. A tabulation format was chosen to describe the characteristics of included studies, the characteristics of participants, and the objectives and outcomes related to the associations between sedentary behavior and physical inactivity with NMS.

### 2.5. Quality assessment

After retrieving the studies that met inclusion criteria, two authors independently assessed the methodological quality of studies using Newcastle-Ottawa Scale [23, 24].

For the longitudinal studies, Newcastle-Ottawa scale for cohort studies was used. For cross sectional studies, quality appraisal was performed using the Newcastle-Ottawa scale adapted for cross-sectional studies [24, 25]. Studies were scored based on information provided on the selection of participants (maximum of 3 points], information about confounders that were controlled for in the studies (maximum of 2 points), and assessment of outcome (maximum of 3 points). Using the summed scores for each study, we categorized the studies as low quality (0–4), moderate quality (5–6), and high quality (≥ 7) [26, 27].

## 3. Results

**Fig 1:**
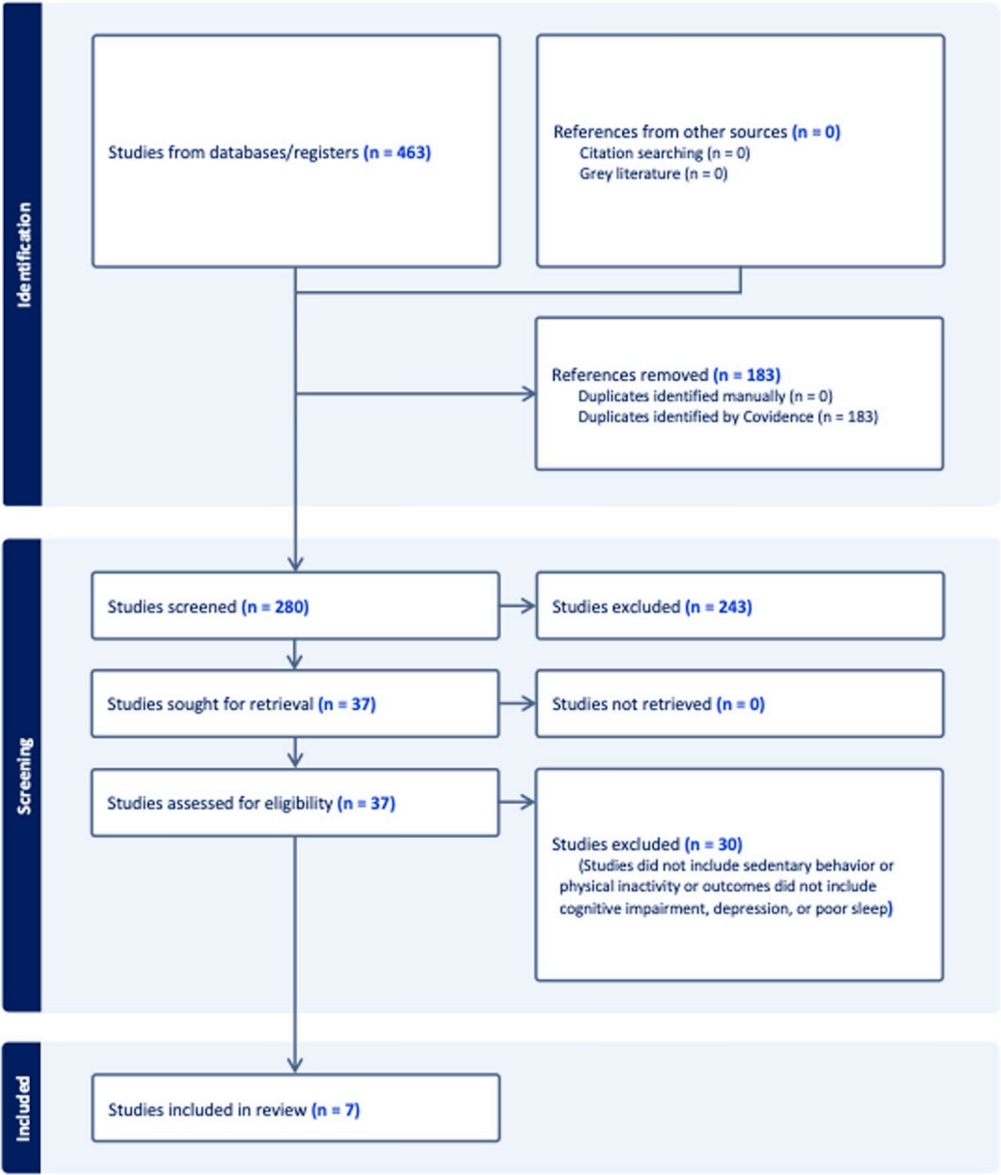
PRISMA flowchart.

Out of 463 articles, 183 duplicate articles were removed, leaving 280 studies. Of these, 243 were considered irrelevant, and 37 studies underwent full-text screening. Finally, 7 studies [15, 28–33] met the eligibility criteria for this systematic review and were included in the data extraction and narrative synthesis. The selection process is visually represented in a PRISMA flowchart (Fig 1).

The characteristics of the included studies are described in Table 1. All seven included studies were observational studies: three employed a cross-sectional design [15, 28, 33], another three utilized a longitudinal cohort design [29, 30, 32], and one was an exploratory observational study [31]. Five studies were conducted in the USA [15, 29, 30, 32, 33], and the remaining two were done in Europe—the United Kingdom [28] and Germany [31]. Five studies assessed sedentary behavior or physical inactivity with sensor-based measures [15, 28, 31–33], while two studies used self-report Physical Activity Scale for the Elderly (PASE) to assess physical inactivity[29, 30]. Six studies [15, 28–31, 33], assessed the association between sedentary behavior or physical inactivity with cognition, while three studies [28, 30, 31] included an assessment of depression in addition to cognition. Only one study [32] studied the link between sedentary behavior and sleep. All the studies were conducted in the community or home-based settings.

**Table 1:** Study characteristics.

First author, publication year, study design	Number of participants, age, sex (male to female percentage), country	Objective	Sedentary behavior/ physical inactivity (exposure and length of assessment)	Non-motor symptoms studied (including domains where applicable)/tools used for assessment	Results
Ellingson et al., 2019 [15] Cross-sectional	52 participants, mean age 67.8 ± 7.9 years, 56% males, 44% females, USA	To investigate the relationships between sedentary behavior and markers of quality of life including a subscale for cognitive function in PD	Objectively measured sedentary time using activPAL and Actigraph accelerometers. 4 valid days (≥10h/d of accelerometer wear time), including 1 weekend day included in the analysis. Self-reported sedentary time measured using Sedentary Behavior Questionnaire (SBQ).	Cognition (memory, executive function, and processing speed): PDQ-39 for quality of life with subscale for cognitive function. The cognitive domain consists of 4 items (items 30–33 on the PDQ-39).	Higher amounts of objectively measured sedentary time accumulated in prolonged bouts were reported to be associated with worse scores on the PDQ summary index (ρ = 0.34; *P* < .05) and on subscales of mobility, cognition, and communication (ρ_range_ = 0.32–0.41; *P* < .05). Higher amounts of self-reported time spent watching television were associated with lower PDQ index score (ρ = 0.35; *P* < .05) as well as for subscales of mobility, emotion, social, cognitive, and communication (ρ_range_ = 0.29–0.39; *P* < .05).
van Uem et al., 2018 [28] Cross sectional	47 participants, median age 70 [65–74] years, 74% males, 26% females, United Kingdom	To investigate the association between amount of physical activity, severity of depression, cognitive function and quality of life in Parkinson’s disease	DynaPort MiniMod accelerometer was used to objectively measure movement data (which includes sedentary behavior called ‘sedentary episodes’ in this study–lying/sitting. 3 consecutive days of accelerometer wear.	Depression: Geriatric Depression Scale, Cognition: Mini-Mental State Examination (MMSE) for cognitive impairment, German version of the Parkinson’s Disease Questionnaire (PDQ)-39 to assess overall quality of life.	Prolonged sedentary bouts were associated with lower quality of life measured with PDQ-39-measured health-related quality of life. Depression measured by Geriatric Depression Scale reported to be a significant predictor of overall quality of life measured with the German version of the PDQ-39, with sedentary bout length being a modifier. Depression (standardized Beta 0.32; p = 0.009), MMSE (standardized Beta -0.25; p = 0.02) alongside motor scores and steps explained 60.5% variance in PDQ-Activities of Daily Living (ADL). Similarly, depression (standardized Beta 0.59; p<0.01), mean sedentary bout length (standardized Beta (0.42; p<0.01), and age explained 60.2% of the variance in PDQ-ADL.
Jones et al., 2020 [29] Longitudinal cohort study	307 participants, mean age 61 ± 9.9 years, 65.8% males, 34.2% females, USA	To investigate the association between participating in everyday physical activity and clinical cognitive outcomes	Subjective measurement of physical (in)activity using self-report *Physical Activity Scale for the Elderly (PASE)*. Follow up for 3 years.	Cognition: memory (Hopkins Verbal Learning Test-Revised), visuospatial abilities (Judgment of Line Orientation), processing speed-attention (Symbols Digits Modalities Test), and language/semantic fluency (Animal Fluency).	Cognitive status was associated with household physical (in)activity. Engaging in less physically-demanding household activities (Beta -0.38; p = 0.002) was associated with a higher risk of PD-related mild cognitive impairment or dementia.
Timblin et al., 2022 [30] Longitudinal cohort study	487 participants, mean age 61.1 ± 9.7 years, 65.1% males, 34.9% females, USA	To investigate the long-term role physical activity between depressive symptoms and cognition in individuals with Parkinson’s disease	Subjective measurement of physical (in)activity *with self-report Physical Activity Scale for the Elderly (PASE)*. Follow up for 5 years.	Cognition: memory (Hopkins Verbal Learning Test-Revised), visuospatial abilities (Judgment of Line Orientation), processing speed-attention (Symbols Digits Modalities Test), and language/semantic fluency (Animal Fluency), and executive function/working memory (Letter-Number Sequencing). Depression: Geriatric Depression Scale-Short Form.	Results suggest that physical activity may be a mediator between depression and cognitive functioning in individuals diagnosed with PD. Depression is more likely to be associated with inactivity in household activities (Beta -0.52; p <0.01), which is indirectly linked to cognitive decline (Beta -0.63; p = 0.017).
Troutman et., 2020 [33] Cross-sectional study	17 participants, mean age 65.1 years, 82% males, 18% females, USA	To investigate the relationship between sedentary time and cognitive performance in individuals with mild-to-moderate Parkinson’s disease	Objective measurement of sedentary behavior using Sensewear pro armband. Percent of waking hours spent sedentary was derived. 72 hours of accelerometer wear.	Cognition (memory, working memory, verbal fluency, and attention): Parkinson’s Disease-Cognitive Rating Scale (PD-CRS) with average of the memory composite and attention scores to create a measure of global cognition, and a computerized task-switching paradigm to measure cognitive flexibility.	The percentage of awake time spent in sedentary activities was negatively associated with attention (Beta = -14.20; p = 0.03), after controlling for moderate-to-vigorous physical activity. The associations were not significant for other cognitive domains.
Sulzer et al., 2021 [31] Longitudinal cohort study	20 participants, median age 67.5 [44–80], 65% males, 35% females, Germany	To investigate the long-term impact of sedentary behavior and cognitive impairment in the home environment and its association to sickness and death in PD	DynaPort Minimod accelerometer was used for objective measurement of sedentary behavior (lying and sitting periods were combined and defined as sedentary behavior). 4.3 years follow up, accelerometer was worn for 72 hours at baseline.	Cognition (memory, verbal fluency, visuospatial task, working memory and attention): Parkinson Neuropsychometric Dementia Assessment (PANDA). Depression: Geriatric Depression Scale (GDS)	Longer sedentary mean bout length (p = 0.02) and cognitive impairment (p<0.01) were associated with health-related study attrition due to sickness and death in PD. Depression was weakly associated with predicted future dropout (Beta 0.13; p = 0.05).
Prusynski et al., 2022 [32] Prospective observational study	25 participants, mean age 69.0 ± 6.0 years, sex not specified, USA	To examine the association between sleep and physical activity in PD and healthy older adults	Objective measurement of sedentary behavior, physical activity and sleep using Fitbit Charge HR activity monitor. 14 days of monitor wear.	Sleep: Total minutes of nighttime sleep. Number of nighttime awakenings (NWAK). Wake time after sleep onset (WASO). Total minutes of daytime sleep. Total number of daytime nap count.	Each additional 30 minutes of nighttime sleep was associated with 25 fewer sedentary minutes in people with PD (Beta -25; p<0.01).

The total number of participants across all studies was 980 (excluding controls), with sample sizes ranging from 17 [33] to 487 [30] participants. Notably, all the studies were conducted within the last decade, reflecting the emerging trend in research to better understand the relationship between sedentary behavior and physical inactivity with NMS in PD. Four studies considered the Hoehn and Yahr staging of PD [28, 31–33]. These studies reported that participants had mild to moderate PD symptoms with minimal functional impairments. For instance, Van Uem et al. [28] reported that only 8% of participants were at Hoehn and Yahr stages 4 or 5. The authors further classified participants with no dementia vs PD dementia and reported that 2/47 participants (stage 4) and 0/47 (stage 5) had no dementia, whereas only 1 participant each at stages 4 and 5 had dementia [24]. In the other studies, Sulzer et al. [31] reported that 11% of participants were at stages 4 or 5, Troutman et al. [33] included only participants at stages 1–2, while Prusynski et al. [32] reported a median of stage 1 for all included participants.

### 3.1 Associations between sedentary behavior and physical inactivity with cognitive impairments in PD

It was consistently reported by six studies that sedentary behavior or physical inactivity were associated with cognitive impairment in PD [15, 28–33]. Interesting variations were observed in the aspects of this relationship investigated by the different studies. One study found that as leisure/recreational physical activities declined over time, there was an association between physical inactivity and an elevated risk of PD-related mild cognitive impairment and dementia [29]. Another study reported that sedentary behavior accumulated in prolonged bouts was associated with overall poor health in individuals with PD, particularly worse in those with cognitive impairment [31]. A study by Troutman et al. reported that higher sedentary behavior was negatively associated with worse attention, after controlling for moderate-to-vigorous physical activity [33]. Taken together, the studies suggest that sedentary behavior and physical inactivity are associated with worse cognition in PD.

### 3.2 Associations between sedentary behavior and physical inactivity with depression in PD

Three studies assessed depression in addition to cognitive impairment in individuals with PD [28, 30]. One study found that depression was associated with prolonged sedentary bout length, which in turn was associated with lower health-related quality of life [28]. In another study, it was reported that physical inactivity was associated with depression, such that individuals with PD who experienced higher depression were more likely to be less physically active, which was indirectly associated with cognitive impairment [30]. A third study reported that prolonged bouts of sedentary behavior and higher depression in PD were associated with higher rates of attrition/drop out from study follow up due to illness or death [28].

### 3.3 Associations between sedentary behavior and physical inactivity with sleep in PD

Only one study assessed the association between sleep and sedentary behavior or physical inactivity in PD [32]. The study reported that individuals with mild PD slept less and were less active compared to a group of healthy older adults. Of note, in both groups, less sleep was associated with more sedentary behavior [32].

### 3.4 Quality appraisal

Using the Newcastle-Ottawa Scale [23, 26], six [15, 28–31, 33] out of the seven studies were of moderate quality (score of 5–6), while one study [32] was rated as low quality (score 0–4), as shown in Table 2. Among the six studies rated as moderate quality, three were cross-sectional [15, 28, 33], while the remaining three were longitudinal studies [29–31]. The most common reasons for a low rating were related to poor description of the non-exposed cohort, lack of control for confounders, sample size justification, and response rate. The specific results of the methodological quality assessments can be found in S3 and S4 Tables.

**Table 2:** Measures and methods used to classify sedentary behavior, physical inactivity, and non-motor symptoms.

Publication	Study measures (sedentary behavior, physical (in)activity, and non-motor symptoms)	Measurement properties	Statistical methods used	Risk of bias score
Ellingson et al., 2019 [15]	*Sedentary behavior* Objectively measured sedentary time using activPAL and Actigraph accelerometers Self-reported sedentary time measured using Sedentary Behavior Questionnaire (SBQ). *Non-motor symptoms* PDQ-39 (cognitive domain)	Actigraph GT3X+ counts per second of each of the 3 axes were utilized. The specific cut-point used is unknown. ActivPAL’s proprietary software used in activity classification with >90% accuracy in classifying sedentary from non-sedentary behaviors [52]. The SBQ has acceptable test-retest reliability (ICC 0.51–0.93) [53]. Significant but small correlation between the PDQ-39 Cognitions score and the neurocognitive composite scores for delayed episodic memory (r = −0.13, p = 0.01) and processing speed (r = −0.12, p = .02) [29].	Spearman’s correlations and linear regression analysis	5
van Uem et al., 2018 [28]	*Sedentary behavior* Objectively measured sedentary behaviour using DynaPort MiniMod accelerometer *Non-motor symptoms* Geriatric Depression Scale PDQ-39 (cognitive domain) Mini-Mental State Examination (MMSE)	A minimum of 2 days of activity monitoring is required to obtain an ICC ≥ 0.7 for most activities [54]. Significant correlation with Hamilton Depression Rating Scale (r = 0.82 P<0.01) [55, 56]. The PDQ-39 correlates significantly with MMSE (r = -0.32, P<0.01) [57]. The PDQ-39 also correlates with the neurocognitive composite scores for delayed episodic memory (r = −0.13, p = 0.01) and processing speed (r = −0.12, p = .02).	Stepwise multivariate regression analyses	5
Jones et al., 2020 [29]	*Physical (in)activity* *Physical Activity Scale for the Elderly (PASE)* *Non-motor symptoms* Hopkins Verbal Learning Test-Revised, Judgment of Line Orientation, Symbols Digits Modalities Test, and Animal Fluency Test	Test-retest reliability (intraclass correlation coefficient) of 0.69 for household-related physical activity [58]. Hopkins Verbal Learning Test- Revised has a reliability of r = 0.74, P<0.01 for total recall, and predicts cognitive decline in PD (hazard ratio (HR) 0.98, P<0.01) [59]. Judgement of Line Orientation Test has a test-retest reliability of 0.90 with standard error of measurement of 1.8 points [60]. Symbol Digit Modalities Test predicts cognitive decline in PD (HR 0.98, P = 0.04) [61]. Animal Fluency is sensitive (0.88) and specific (0.96) in early detection of dementia [62].	Ordinal multilevel modeling (MLM)	5
Timblin et al., 2022 [30]	*Physical (in)activity* *Physical Activity Scale for the Elderly (PASE)* [58] *Non-motor symptoms* Hopkins Verbal Learning Test- Revised, Judgement of Line Orientation Test, Letter-Number Sequencing task, Symbols Digits Modalities Test, and Animal Fluency. Geriatric Depression Scale [55, 56]	Reported above. In addition to measurement properties reported above, the Letter-Number Sequencing task has good test-retest reliability (ICC = 0.64) in PD. Reported above.	Structural equation modeling (SEM)	5
Troutman et al., 2020 [33]	*Sedentary behavior* Objective measurement of sedentary behavior using Sensewear pro armband *Non-motor symptoms* Cognition: Parkinson’s Disease-Cognitive Rating Scale (PD-CRS)	At least 3 weekdays of monitoring required to achieve a reliability of 0.80 [63]. The PD-CRS has a test-retest reliability (ICC) >0.70, and sensitive (94%) and specific (94%) to detect PD dementia [54, 64].	Linear regression	6
Sulzer et al., 2021 [31] Longitudinal cohort study	*Sedentary behavior* Objectively measured sedentary behavior using DynaPort MiniMod accelerometer [54] *Non-motor symptoms* Cognition: Parkinson Neuropsychometric Dementia Assessment (PANDA). Geriatric Depression Scale [55, 56]	Reported above. The PANDA (cognition) had a specificity of 91% and a sensitivity of 90% for PD dementia and 77% for PD dementia plus PD-mild cognitive disorder [65]. Reported above.	Binary logistic regression	6
Prusynski et al., 2022 [32] Prospective observational study	*Sedentary behavior/Non-motor symptoms* Objective measurement of sedentary behavior, physical activity and sleep using Fitbit Charge HR activity monitor.	Fitbit devices can correctly identify sleep epochs with accuracy of 0.81 to 0.91, sensitivity of 0.87 and 0.99, and specificity of 0.10 and 0.52 [66].	Linear regression	3

## 4. Discussion

### 4.1. Summary of key findings

This review synthesized evidence on the associations between sedentary behavior, physical inactivity, and NMS namely cognitive impairment, depression and sleep in PD. Our results show that higher sedentary behavior, especially when accumulated in prolonged bouts, is associated with cognitive impairment in PD. Physical inactivity was also associated with a higher risk for PD-related cognitive impairment. A bidirectional relationship appears to exist between depression and sedentary behavior, where depression is linked to prolonged sedentary bouts and vice versa, while physical inactivity is associated with higher depression levels but then indirectly linked to cognitive impairment. Also, there is evidence that higher sedentary behavior is associated with shorter sleep duration. The variability in the measurement tools for sedentary behavior and physical inactivity as well as NMS is a major barrier to determining the precise magnitude of the associations. Five studies used device-based measures of sedentary behavior or physical inactivity [15, 28, 31, 32] whereas two studies used self-report measures of physical (in)activity [29, 30]. Furthermore, one of the three cross-sectional studies assessed cognition using the 4-item subscale from the PDQ-39 [28], which may not capture the full spectrum of cognitive impairments in PD nor correlates well with standard neuropsychological tests [34].

There is some evidence that physical activity is highly beneficial for individuals with PD, potentially delaying or slowing down the disease progression [35, 36]. Exercise has also been demonstrated to improve NMS of PD [37]. However, it is important to recognize that sedentary behavior is not merely the opposite of physical activity or exercise, but an independent behavior that is associated with adverse health outcomes[15, 16]. The unique relationship between sedentary behavior, physical inactivity, and NMS in PD has been suggested by some researchers [15, 38]. Yet, a clearer understanding of this relationship is required, considering that the prevalence of PD is increasing, and technological advances may encourage even more sedentary behaviors. Although this gap has been highlighted in recent years [39], a better understanding of the associations between sedentary behavior, physical inactivity, and NMS is needed.

### 4.2. Associations between sedentary behavior and physical inactivity with cognitive impairments in PD

Cognitive impairment is a common NMS in PD, with a wide spectrum of symptoms related to attention, working memory, visuospatial, and executive functioning [40, 41]. The underlying mechanisms contributing to cognitive impairment in PD are complex and may involve genetic factors such as α-synuclein toxicity, Lewy body accumulation, synaptic changes, inflammatory process, and neurotransmitter disruptions [40–43]. Pharmacological interventions are typically used to target these changes in order to enhance cognition, however, the overall improvement remains modest [41]. In addition to pharmacological interventions, non-pharmacological strategies, such as physical exercise or cognitive training, have shown some promise in improving NMS in PD [40, 41, 44]. Although most studies have focused on physical activity and exercise, in the general population, there is evidence from a systematic review of the associations between higher sedentary behavior and impaired cognitive function across the lifespan [45]. Importantly, such associations have been suggested in PD, though less studied [15], the evidence from this review shows that sedentary behavior is associated with cognitive impairment in PD.

### 4.3. Associations between sedentary behavior and physical inactivity with depression in PD

Another NMS less studied in PD is depression and its association with sedentary behavior and physical inactivity. The combined findings from the 3 studies that assessed depression in this review underscore the complex relationship between depression, sedentary behavior, and health-related quality of life in PD. Prolonged sedentary behaviour was associated with depression and worsening depression over time was linked with reduced engagement in household activities, which correlated, with declines in global cognition. Furthermore, the impact of prolonged sedentary behavior and higher depression in PD extended to higher rates of attrition and dropout from study follow-up due to illness or death, emphasizing the critical role of mental health and activity levels in study participation and overall outcomes for individuals with PD. Similar findings with a study that evaluated the predictors of physical activity in PD showed that depression and balance impairments were the most significant factors [46]. Other studies have also demonstrated a link between physical inactivity and an increased likelihood of depression in PD [16, 30, 37]. Depression often manifests before the onset of motor symptoms, though it can occur at any stage of PD [47, 48]. Approximately 40% of patients with PD experience symptoms of depression [48]. These symptoms may include excessive feeling of sadness, helplessness, lack of concentration, loss of interest in previously enjoyed activities, increased exhaustion, irritability, and dysphoria. Diagnosing depression in PD can be challenging due to symptom overlap with other PD symptoms, leading to the condition being untreated in some individuals living with PD [47, 48]. Depression is not only associated with emotional burden, but may have a significant negative impact on quality of life [48]. Moreover, depression also negatively affects motor symptoms, and worsens cognitive and functional disabilities [28, 48]. It is noteworthy that depression along with anxiety are among the NMS that are considered to be the most important predictors of quality of life in individuals with PD [28, 49].

### 4.4. Associations between sedentary behavior and physical inactivity with sleep in PD

Only one study assessed sleep in this review and demonstrated an independent relationship between less sleep and higher sedentary behavior in PD [32]. It has been suggested that treating sleep deficits may lead to a decline in sedentary behavior in the early stages of PD [5]. This indicates the potential bidirectional relationship between sleep and sedentary behavior in PD. Although research on this topic is limited, further studies will be required to explore this relationship.

### 4.5. Limitations

It is important to note that the evidence provided in this paper is not without limitations. A meta-analysis was not performed due to the small number of studies, heterogeneity of methods across the studies in assessments of sedentary behavior, physical inactivity, and NMS. As this is an emerging area of research, we included a broad search strategy, which included terms with relatively close meaning to sedentary behavior or physical inactivity such as ‘in-bed’. Two cross-sectional studies assessed cognition using the 4-item cognitive domain subscale of the PDQ-39 which is not a standardized test for cognition. Physical inactivity was assessed in 2 studies using a self-report tool, which may be subject to recall bias. Future studies should consider using standardized assessments for NMS and include objective measurements of sedentary behavior and physical (in)activity to provide clear evidence that could guide intervention studies. Furthermore, studies included in this paper were collected from peer-reviewed journals via electronic databases. Thus, studies selected for this paper could potentially be subject to publication bias. Finally, all the studies were observational and no causal relationships between sedentary behavior, physical inactivity, with NMS can be inferred from this review.

## 5. Implications of the results for practice, policy, and future research

It is well established that NMS of PD are a major contributor to the reduced quality of life in individuals living with PD [3, 43]. Despite often preceding the motor symptoms, NMS have not received sufficient attention and remain undertreated [50]. Currently, various pharmacological as well as non-pharmacological treatments are being investigated for both motor and NMS of PD [51]. Exercise has emerged as a promising non-pharmacological approach for improving not only motor symptoms but also NMS [16]. However, it is essential to recognize that sedentary behavior is an independent risk factor for poor health outcomes [19]. Individuals with PD often lead sedentary lifestyles, making it crucial to assess the association with NMS in PD in more depth [15]. Emerging research has highlighted the unique and crucial role of sedentary behavior and physical inactivity in PD [13]. For example, it has been demonstrated that individuals with PD tend to have longer bouts of sedentary behavior compared to their age-matched controls, suggesting a clear change in their pattern of sedentary behavior [49]. Despite its potential significance, current data on the role of sedentary behavior in PD, particularly in the context of NMS, is severely limited. Only 7 studies were found relevant to our topic, indicating the need for further investigation in this area. A clearer understanding of the relationship between sedentary behavior and NMS may help to establish better behavioral markers of the disease at an earlier stage, leading to more effective therapies for PD.

There are some implications of our findings for clinical practice, policy, and future research. Incorporating objective measures of physical inactivity and sedentary behavior into clinical assessments may help to develop more precise rehabilitation strategies to help with motor and NMS. Healthcare professionals should prioritize interventions aimed at frequently interrupting sedentary behavior throughout waking hours considering the high prevalence sedentary behavior in people with PD [38]. Such interventions not only hold promise for mitigating NMS but also for improving cardiometabolic health and preventing secondary comorbidities [13, 19].

Additionally, investigating the effectiveness of interventions aimed at reducing sedentary behavior and mitigating physical inactivity and their associations with cognitive impairment, depression, and sleep disturbances in PD patients would provide valuable insights for clinical practice and policy formulation. Future studies with larger sample sizes and improved study designs, adhering closely to the current definition of sedentary behavior [14] are warranted to offer better insight into the associations of sedentary behavior and/or physical inactivity with NMS in PD.

In conclusion, while exercise has shown promise in addressing motor and NMS in PD, the specific impact of sedentary behavior and physical inactivity on the disease process remains an important area for further research. Understanding the role of reducing sedentary behavior and physical inactivity in PD may open new avenues for therapeutic interventions and lead to improved outcomes for individuals living with the disease.

## Supporting information

S1 Table PRISMA 2020 checklist. (DOCX)

S2 Table Data extraction form. (DOCX)

S3 Table Quality appraisal of longitudinal studies using Newcastle Ottawa Scale. (DOCX)

S4 Table Quality appraisal of cross-sectional studies using the adapted Newcastle Ottawa Scale for case-control studies. (DOCX)
