# Supplementary material for: A systematic review of the associations between sedentary behavior, physical inactivity, and non-motor symptoms of Parkinson’s disease
Source: PLoS One. 2024 Mar 29;19(3):e0293382. doi: 10.1371/journal.pone.0293382 (PMC10980241; doi:10.1371/journal.pone.0293382)
Supplement: S2 Table — (DOCX) [file pone.0293382.s002.docx]

**S2 Table**: Data extraction form

**Study 1**: Ellingson LD, Zaman A, Stegemöller EL. Sedentary Behavior and Quality of Life in Individuals With Parkinson's Disease. Neurorehabil Neural Repair. 2019;33(8):595-601.

| **Author**: Ellingson et al.  **Title**: Sedentary Behavior and Quality of Life in Individuals With Parkinson’s Disease  **Year**: 2019  **Country**: United States of America  **Age**: Mean age: 67.8 years; SD 7.9  **Sex** (%): 56% males, 44% females  **Study design**: Cross-sectional  **Objective(s) of study**: To investigate the relationships between sedentary behavior and markers of quality of life including a subscale for cognitive function in PD  **Number of participants**: 52 participants  **Type of non-motor symptom**: Cognition  **Means of measuring inactivity or sedentary behavior**: ActivPAL and Actigraph; Sedentary Behavior Questionnaire (SBQ).  **Measurement duration**: 4 valid days (≥10h/d of wear time), including 1 weekend day  **Means of measuring motor symptom (if measured**): The Unified Parkinson’s Disease Rating Scale (UPDRS)  **Tools used for outcome assessment**: Cognition: PDQ-39 for quality of life with subscale for cognitive function. The cognitive domain consists of 4 items (items 30–33 on the PDQ-39  **Key findings and statistical analysis**: Higher amounts of objectively measured sedentary time accumulated in prolonged bouts were reported to be associated with worse scores on the PDQ summary index (ρ = 0.34; P < .05) and on subscales of mobility, cognition, and communication (ρrange = 0.32-0.41; P < .05).  Higher amounts of self-reported time spent watching television were associated with lower PDQ index score (ρ = 0.35; P < .05) as well as for subscales of mobility, emotion, social, cognitive, and communication (ρrange = 0.29-0.39; P < .05). Spearman’s correlations and linear regression analysis. |
| --- |

| **Study 2:** van Uem JMT, Cerff B, Kampmeyer M, Prinzen J, Zuidema M, Hobert MA, Gräber S, Berg D, Maetzler W, Liepelt-Scarfone I. The association between objectively measured physical activity, depression, cognition, and health-related quality of life in Parkinson's disease. Parkinsonism Relat Disord. 2018;48:74-81. |
| --- |
| **Author**: van Uem et al.  **Title**: The association between objectively measured physical activity, depression, cognition, and health-related quality of life in Parkinson's disease  **Year**: 2018  **Country**: United Kingdom  **Age**: Median age: 70 years IQR[65-74]  **Sex** (%): 74% males, 26% females  **Study design**: Cross-sectional  **Objective(s) of study**: To investigate the association between amount of physical activity, severity of depression, cognitive function and quality of life in Parkinson's disease  **Number of participants**: 47 participants  **Type of non-motor symptom**: Depression, cognition  **Means of measuring inactivity or sedentary behavior**: DynaPort MiniMod accelerometer  **Measurement duration**: 3 consecutive days of wear  **Means of measuring motor symptom (if measured**): The Unified Parkinson’s Disease Rating Scale (UPDRS)-Part III  **Tools used for outcome assessment**: Depression: Geriatric Depression Scale; Cognition: Mini-Mental Status Exam (MMSE); Quality of Life: PDQ-39.  **Key findings and statistical analysis**: Prolonged sedentary behavior was associated with worse quality of life. Depression (standardized Beta 0.32; p=0.009), MMSE (standardized Beta -0.25; p=0.02) alongside motor scores and steps explained 60.5% variance in PDQ-Activities of Daily Living (ADL). Similarly, depression (standardized Beta 0.59; p<0.01), mean sedentary bout length (standardized Beta (0.42; p<0.01), and age explained 60.2% of the variance in PDQ-ADL. Stepwise multivariate regression analyses. |

| **Study 3.** Jones JD, Baxter F, Timblin H, Rivas R, Hill CR. Physical inactivity is associated with Parkinson’s disease mild cognitive impairment and dementia. Ment Health Phys Act. 2022;23:100461 |
| --- |
| **Author**: Jones et al.  **Title**: Physical inactivity is associated with Parkinson's disease mild cognitive impairment and dementia  **Year**: 2022  **Country**: United States of America  **Age**: Mean age: 61 years; SD: 9.9  **Sex** (%): 65.8% males, 34.2% females  **Study design**: Longitudinal cohort study.  **Objective(s) of study**: To investigate the association between participating in everyday physical activity and cognitive outcomes.  **Number of participants**: 307 participants  **Type of non-motor symptom**: Cognition  **Means of measuring inactivity or sedentary behavior**: Physical Activity Scale for the Elderly (PASE).  **Measurement duration**: Follow up for 3 years.  **Means of measuring motor symptom (if measured**): The Unified Parkinson's Disease Rating Scale-part III (UPDRS-III).  **Tools used for outcome assessment**: Cognitive status assessed with the Hopkins Verbal Learning Test-Revised, Judgment of Line Orientation, Symbols Digits Modalities Test, and Animal Fluency.  **Key findings and statistical analysis**: Cognitive status was associated with household physical (in)activity. Engaging in less physically-demanding household activities (Beta -0.38; p=0.002) was associated with a higher risk of PD-related mild cognitive impairment or dementia. Ordinal multilevel modeling (MLM). |

| **Study 4.** Timblin H, Rahmani E, Ryczek CA, Hill CR, Jones JD. Physical inactivity links depressive symptoms and cognitive functioning among individuals with Parkinson's disease. Neuropsychology. 2022;36(6):505-512 |
| --- |
| **Author**: Timblin et al.  **Title**: Physical inactivity links depressive symptoms and cognitive functioning among individuals with Parkinson's disease  **Year**: 2022  **Country**: United States of America  **Age**: Mean age: 61.1 years; SD: 9.7  **Sex** (%): 65.1% males, 34.9 % females  **Study design**: Longitudinal cohort study  **Objective(s) of study**: To investigate the long-term role physical activity between depressive symptoms and cognition in individuals with Parkinson's disease  **Number of participants**: 487 participants  **Types of non-motor symptoms:** Cognition and Depression  **Means of measuring inactivity or sedentary behavior**: Physical Activity Scale for the Elderly (PASE).  **Measurement duration**: Follow up for 5 years  **Means of measuring motor symptom (if measured**): The Unified Parkinson's Disease Rating Scale-part III (UPDRS-III)  **Tools used for outcome assessment**: Cognition: Hopkins Verbal Learning Test- Revised, Judgement of Line Orientation Test, Letter-Number Sequencing task, Symbols Digits Modalities Test, and Animal Fluency.  Depression: Geriatric Depression Scale-Short Form.  **Key findings and statistical analysis**: Results suggest that physical activity may be a mediator between depression and cognitive functioning in individuals diagnosed with PD. Depression is more likely to be associated with inactivity in household activities (Beta -0.52; p <0.01), which is indirectly linked to cognitive decline (Beta -0.63; p = 0.017). Structural equation modeling (SEM). |

| **Study 5.** Troutman SBW, Erickson KI, Grove G, Weinstein AM. Sedentary Time is Associated with Worse Attention in Parkinson's Disease: A Pilot Study. J Mov Disord. 2020;13(2):146-149 |
| --- |
| **Author**: Troutman et al.  **Title**: Sedentary Time is Associated with Worse Attention in Parkinson's Disease  **Year**: 2020  **Country**: United States of America  **Age**: Mean age 65.1 years    **Sex** (%): 82% males, 18% females  **Study design**: Cross-sectional study  **Objective(s) of study**: To investigate the relationship between sedentary time and cognitive performance in individuals with mild-to-moderate Parkinson's disease  **Number of participants**: 17 participants  **Type of non-motor symptom**: Cognition  **Means of measuring inactivity or sedentary behavior**: Sensewear pro armband  **Measurement duration**: 72 hours  **Means of measuring motor symptom (if measured**): The Unified Parkinson's Disease Rating Scale-motor score  **Tools used for outcome assessment**: Cognition: Parkinson’s Disease-Cognitive Rating Scale (PD-CRS).  **Key findings and statistical analysis**: The percentage of awake time spent in sedentary activities was negatively associated with attention (Beta =-14.20; p=0.03), after controlling for moderate-to-vigorous physical activity and medication dosage. The associations were not significant for other cognitive domains. Linear regression. |

| **Study 6.** Sulzer P, Gräber S, Schaeffer E, van Lummel R, Berg D, Maetzler W, Liepelt-Scarfone I. Cognitive impairment and sedentary behavior predict health-related attrition in a prospective longitudinal Parkinson's disease study. Parkinsonism Relat Disord. 2021;82:37-43. |
| --- |
| **Author**: Sulzer et al.  **Title**: Cognitive impairment and sedentary behavior predict health-related attrition in a prospective longitudinal Parkinson’s disease study  **Year**: 2021  **Country**: Germany  **Age**: Median age: 67.5 years, IQR [44-80]  **Sex** (%): 65% males, 35% females  **Study design**: Longitudinal cohort study  **Objective(s) of study**: To investigate the long-term impact of sedentary behavior and cognitive impairment in the home environment and its association to sickness and death in PD  **Number of participants**: 20 participants  **Types of non-motor symptoms**: Cognition and Depression  **Means of measuring inactivity or sedentary behavior**: DynaPort Minimod accelerometer  **Measurement duration**: 4.3 years follow up. The Accelerometer was worn for 72 hours at baseline.  **Means of measuring motor symptom (if measured**): The Unified Parkinson's Disease Rating Scale-part III (UPDRS-III)  **Tools used for outcome assessment**: Parkinson Neuropsychometric Dementia Assessment (PANDA) and a neuropsychological test battery to classify cognitive status. Depression assessed using the Geriatric Depression Scale (GDS).  **Key findings and statistical analysis**: longer sedentary mean bout length (p=0.02) and cognitive impairment (p<0.01) were associated with health-related study attrition due to sickness and death in PD. Depression was weakly associated with predicted future dropout (Beta 0.13; p=0.05). Linear regression. |

| **Study 7.** Prusynski RA, Kelly VE, Fogelberg DJ, Pradhan S. The association between sleep deficits and sedentary behavior in people with mild Parkinson disease. Disabil Rehabil. 2022;44(19):5585-5591 |
| --- |
| **Author**: Prusynski et al.  **Title**: The association between sleep deficits and sedentary behavior in people with mild Parkinson disease  **Year**: 2022  **Country**: United States of America  **Age**: Mean age: 69.0 years; SD 6.0  **Sex** (%): not specified  **Study design**: Prospective observational study  **Objective(s) of study**: To examine the association between sleep and physical activity in PD and healthy older adults  **Number of participants**: 25 participants  **Type of non-motor symptom**: Sleep  **Means of measuring inactivity or sedentary behavior**: Fitbit Charge HR  **Measurement duration**: 14 days  **Means of measuring motor symptom (if measured**): The Unified Parkinson's Disease Rating Scale-part III (UPDRS-III)  **Tools used for outcome assessment**: Total minutes of nighttime sleep, number of nighttime awakenings (NWAK), wake time after sleep onset (WASO), total minutes of daytime sleep, and total number of daytime nap count. These measures were derived from the Fitbit based on a proprietary algorithm developed by the device manufacturer that combines actigraphy and heart rate measures.  **Key findings and statistical analysis**: Each additional 30 minutes of nighttime sleep was associated with 25 fewer sedentary minutes in people with PD (Beta -25; p<0.01). Linear regression. |
