# Supplementary material for: A systematic review of the associations between sedentary behavior, physical inactivity, and non-motor symptoms of Parkinson’s disease
Source: PLoS One. 2024 Mar 29;19(3):e0293382. doi: 10.1371/journal.pone.0293382 (PMC10980241; doi:10.1371/journal.pone.0293382)
Supplement: S3 Table — (DOCX) [file pone.0293382.s003.docx]

**S3 Table:** Quality appraisal of longitudinal studies using Newcastle Ottawa Scale (NOS)

| **First author, publication year** | **Selection** | | | | **Comparability** | **Outcome** | | | **Total** |
| --- | --- | --- | --- | --- | --- | --- | --- | --- | --- |
|  | Representativeness of exposed cohort | Selection of the non-exposed cohort | Ascertainment of exposure: | Demonstration that outcome of interest was not present at start of study |  | Assessment of outcome: | Was follow-up long enough for outcomes to occur | Adequacy of follow-up of cohorts |  |
| Jones JD,  2020 | * | - | - | * | - | * | * | * | ***** |
| Timblin H,  2022 | * | - | - | * | - | * | * | * | ***** |
| Sulzer P, 2021 | * | - | * | * | - | * | * | * | ****** |
| Rachel A. Prusynski,  2022 | * | - | - | - | * | * | - | - | *** |

The three main categories in the NOS are: Selection, Comparability and Outcomes with some further subdivisions.

-Selection: exposed cohort representation (*), non-exposed cohort selection (*), exposure ascertainment (*), outcome not present at start of study (*).

-Comparability: Cohort comparability based on the design or analysis controlled for confounders (*).

-Outcome: Assessment of outcome (*), length of follow-up (*), adequacy of follow-up of cohorts (*).

Using the total scores for each study, we categorized the studies as low quality (0-4 stars), moderate quality (5-6 stars), and high quality (≥ 7 stars).

*: star, -: no star
