# Supplementary material for: A systematic review of the associations between sedentary behavior, physical inactivity, and non-motor symptoms of Parkinson’s disease
Source: PLoS One. 2024 Mar 29;19(3):e0293382. doi: 10.1371/journal.pone.0293382 (PMC10980241; doi:10.1371/journal.pone.0293382)
Supplement: S4 Table — (DOCX) [file pone.0293382.s004.docx]

**S4 Table:** Quality appraisal of cross-sectional studies using the adapted Newcastle Ottawa Scale (NOS) for case-control studies

| **First author, publication year** | **Selection** | | | | **Comparability** | **Outcome** | | **Total** |
| --- | --- | --- | --- | --- | --- | --- | --- | --- |
|  | Representativeness of exposed cohort | Sample size | Non-response rate | Ascertainment of the screening/surveillance tool |  | Assessment of outcome | Statistical test |  |
| Ellingson LD,  2019 | * | - | - | ** | - | ** | * | ****** |
| van Uem JMT, 2018 | - | - | - | ** | - | ** | * | ***** |
| Troutman SBW,  2020 | * | - | - | * | - | ** | * | ***** |

The three main categories in the NOS are: Selection, Comparability and Outcomes with some further subdivisions.

-Selection: representative cases (*), justified sample size (*), satisfactory response rate (*), validated tools (2*).

-Comparability: comparable groups and confounding factors are controlled (*).

-Outcome: independent blind assessment (2*), record linkage (2*), self-report (*), appropriate statistical test (*).

Using the total scores for each study, we categorized the studies as low quality (0-4 stars), moderate quality (5-6 stars), and high quality (≥ 7 stars).

*: star, -: no star
